# Associations between triglyceride glucose-body mass index and 30-day mortality in patients with hemorrhagic stroke: analysis of the MIMIC-IV database

**DOI:** 10.3389/fneur.2025.1602822

**Published:** 2025-08-08

**Authors:** Yuexin Lu, Enjie Song, Ruilin Chen, Long Jing, Jiahao Tang, Shunan Shi, Ming Wang, Shu Wan

**Affiliations:** ^1^Brain Center, Zhejiang Hospital, Hangzhou, China; ^2^The Second School of Clinical Medicine, Zhejiang Chinese Medical University, Hangzhou, China; ^3^Zhejiang University, Hangzhou, China; ^4^Zhejiang Province Engineering Research Center for Precision Medicine in Cerebrovascular Diseases, Hangzhou, China

**Keywords:** triglyceride glucose-body mass index, hemorrhagic stroke, all-cause mortality, MIMIC-IV database, intensive care unit

## Abstract

**Background:**

Hemorrhagic stroke (HS), accounting for over 40% of stroke-related deaths, imposes a severe global health burden due to high mortality and disability rates. The triglyceride-glucose-body mass index (TyG-BMI), integrating lipid-glucose metabolism and obesity, has shown prognostic value in cardiovascular diseases but remains underexplored in HS populations.

**Methods:**

This retrospective cohort study analyzed 413 HS patients from the MIMIC-IV database. Multivariable Cox regression, Kaplan–Meier analysis, and restricted cubic spline models assessed mortality risks at 30-day, 90-day, and 1-year intervals. Subgroup analysis and interaction tests were performed to evaluate the robustness of the results.

**Results:**

Compared with the intermediate TyG-BMI group, the low TyG-BMI group exhibited a significantly increased risk of death at 30 days (adjusted hazard ratio [aHR] = 1.836, *p* = 0.010), 90 days (aHR = 1.694, *p* = 0.016), and 1 year (aHR = 1.642, *p* = 0.014). Similarly, the high TyG-BMI group also showed higher mortality risk at 30 days (aHR = 1.584, *p* = 0.039), 90 days (aHR = 1.571, *p* = 0.024), and 1 year (aHR = 1.484, *p* = 0.030). Kaplan–Meier analysis revealed the highest survival rate in the middle tertile group. Restricted cubic spline curve showed a U-shaped relationship emerged between TyG-BMI and mortality. No interactions between TyG-BMI and the stratified variables, except for coronary artery disease.

**Conclusion:**

Patients with intermediate TyG-BMI levels have a lower 30-day mortality risk. As a composite metabolic-nutritional marker, TyG-BMI aids risk stratification in intensive care unit settings.

## Graphical abstract

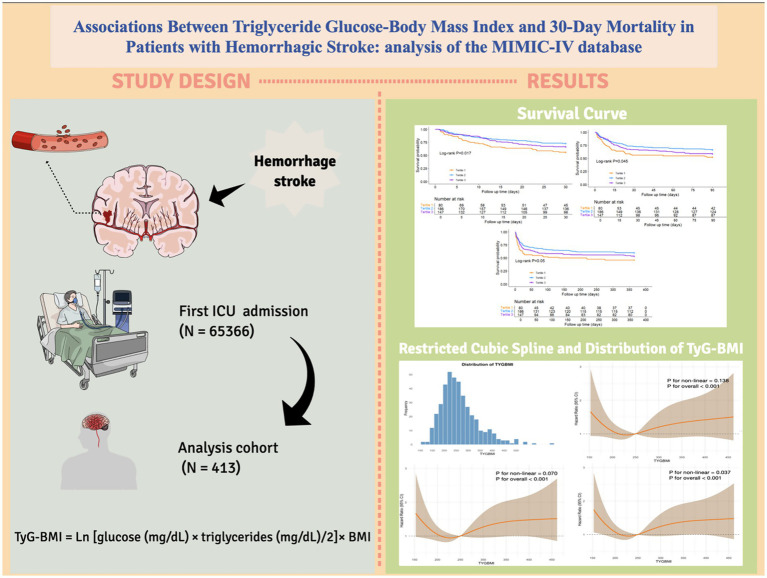

## Introduction

Stroke is a leading cause of death and long-term disability worldwide, placing a substantial burden on global healthcare systems (1, 2). Hemorrhagic stroke (HS), which includes intracerebral hemorrhage (ICH) and spontaneous subarachnoid hemorrhage (SAH), accounts for 27.9 and 9.7% of all stroke cases, respectively (3). However, due to its catastrophic neurological consequences, it is responsible for over 40% of stroke-related deaths (4). Notably, the mortality and disability rates associated with hemorrhagic stroke are significantly higher than those associated with ischemic stroke, especially in low-and middle-income countries (5). This underscores the urgent need for reliable prognostic biomarkers to identify high-risk patients early, optimize treatment allocation, and improve clinical outcomes.

Insulin resistance (IR), characterized by hyperglycemia, dyslipidemia, obesity, and hypertension, is a key metabolic driver of stroke (6). The triglyceride-glucose-body mass index (TyG-BMI), calculated as the product of fasting triglycerides, fasting blood glucose, and body mass index (BMI), integrates lipid metabolism, glucose status, and obesity into a single index. It has been shown to outperform individual parameters in assessing IR and predicting cardiovascular diseases (7). Interestingly, recent studies have reported conflicting findings. In stroke cohorts, lower TyG-BMI has been significantly associated with higher long-term all-cause mortality risk (8), whereas in ischemic stroke cohorts, TyG-BMI has shown a positive correlation with mortality (9). These contradictory observations highlight that the prognostic role of TyG-BMI may depend on specific clinical contexts, warranting disease-specific investigations.

Although the prognostic value of TyG-BMI has been established in ischemic stroke and cardiovascular diseases, its role in hemorrhagic stroke populations remains underexplored, particularly among patients at higher risk of systemic complications such as metabolic instability, and secondary brain injury.

This study, utilizing data from the MIMIC-IV database, aims to assess the association between TyG-BMI and all-cause mortality in HS patients, to establish it as a predictive tool for early risk stratification and personalized interventions to reduce global disease burden.

## Methods

### Data source and data privacy

This retrospective cohort study is based on health-related data obtained from the MIMIC-IV database version 3.0. The MIMIC-IV database contains comprehensive and extensive medical records of patients admitted to the intensive care unit (ICU) at Beth Israel Deaconess Medical Center (BIDMC, Boston, MA, United States) (10). The author, Yuexin Lu, completed the Collaborative Institutional Training Initiative (CITI) program (record ID: 12901560), which granted access to and permission for data extraction from the MIMIC-IV database. Given the deidentified nature of the data, the Institutional Review Board of Beth Israel Deaconess Medical Center waived the requirement for informed consent.

### Study population

**Figure 1:**
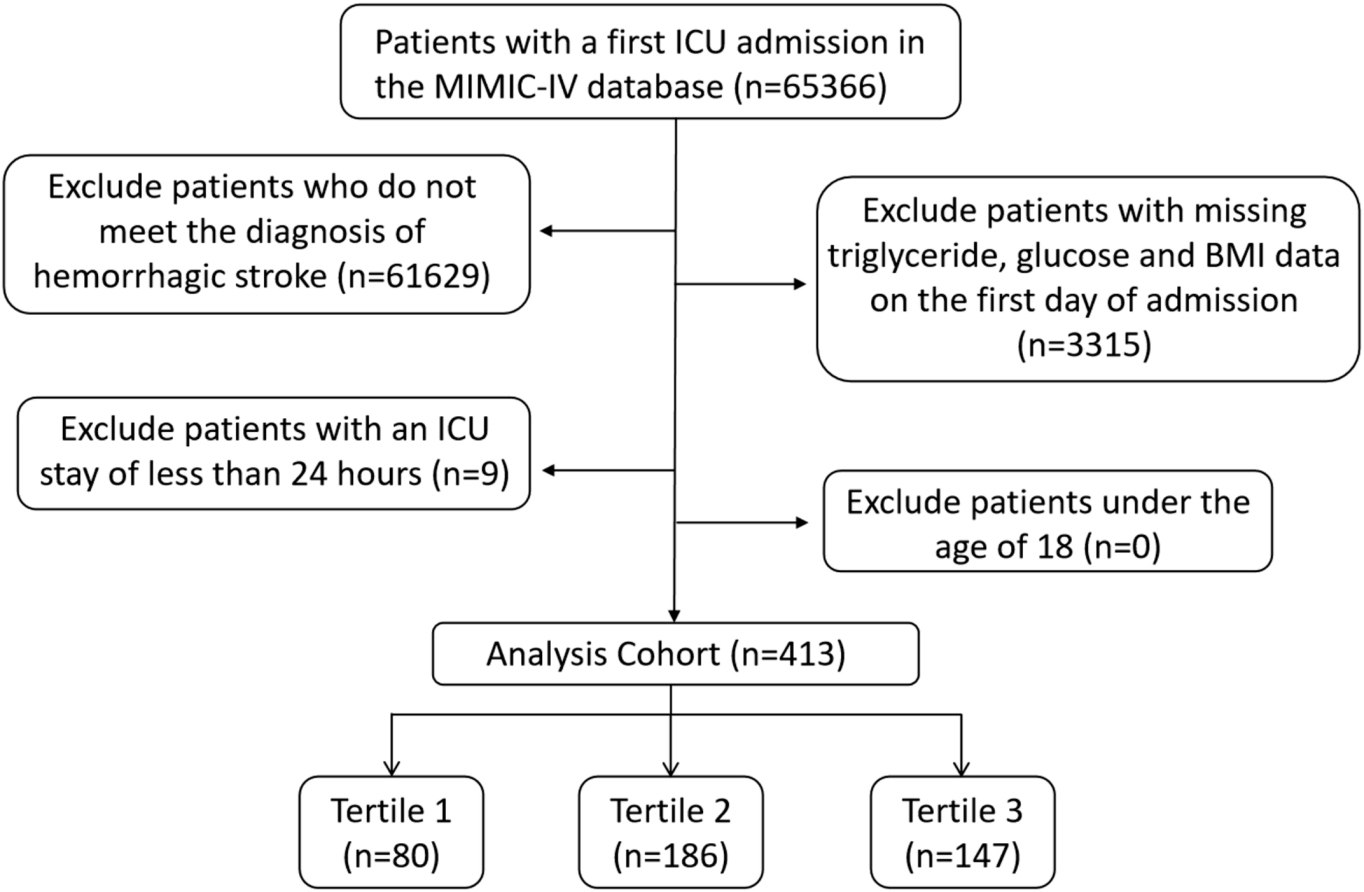
Flowchart of participant selection.

This retrospective study included patients diagnosed with ICH and SAH based on the International Classification of Diseases, 9th and 10th revisions. The exclusion criteria were as follows: (1) patients with multiple ICU admissions due to HS, with data extracted only from their first admission; (2) patients whose key data such as blood glucose, triglycerides, and body mass index were missing; (3) patients with an ICU stay of less than 24 h; and (4) patients under the age of 18 years at the time of first admission. Ultimately, 413 HS patients were included in this study. The inclusion flowchart is shown in Figure 1.

### Data collection and management of missing values

Data were extracted via PostgreSQL 16.4 and Navicat Premium version 17 from the database using Structured Query Language (SQL). Comprehensive data regarding each patient were collected, such as demographic variables: age, gender, race, height, and weight; clinical severity scores at admission: the Glasgow Coma Scale, Sequential Organ Failure Assessment (SOFA) score, and Acute Physiology Score III (APSIII); comorbidities: hypertension, diabetes, chronic obstructive pulmonary disease (COPD), arrhythmia, coronary artery disease (CAD), sepsis, liver disease, hyperlipemia, ventilator-associated pneumonia, Charlson comorbidity index; laboratory markers: triglyceride, glucose, red blood cells, white blood cells, platelets, hemoglobin, creatinine, chloride, albumin, blood urea nitrogen, sodium, potassium, lactate, bicarbonate, prothrombin time and international normalized ratio; treatment methods: includes vasopressors, anticoagulants, antiplatelet drugs, lipid-lowering drugs, and oxygen delivery; clinical outcomes: Length of stay (LOS) in ICU, LOS in hospital; ICU, in-hospital, 30-day; 90-day, 1-year, ICU, in-hospital mortality. All laboratory markers and severity scores were extracted from the first recorded data after ICU admission.

### Outcomes and measures

The primary outcome of this study was 30-day all-cause mortality. While the secondary outcomes included 90-day mortality, 1-year mortality, ICU mortality, and in-hospital mortality.

To minimize potential bias caused by missing data, variables with more than 20% missing values were excluded from the study. The results of the data integrity checks are shown in the Supplementary Table 1. Multiple imputation was performed on variables with less than 20% missing values using the “mice” package in R software.

### TyG-BMI calculation

The TyG was computed as follows: Ln [glucose (mg/dL) × triglycerides (mg/dL)/2] while BMI was calculated by dividing the body weight (kg) by the square of height (m) (kg/m^2^). The TyG-BMI index was calculated as TyG × BMI.

In this study, we grouped the variable TyG-BMI to explore its relationship with survival time and mortality risk. To determine the optimal cutoff point, we utilized the surv_cutpoint function from the R package “survival,” which identifies the best cutoff by maximizing the differences in survival data. After calculating the first optimal cutoff point using this method, to ensure a reasonable sample size for the intermediate group and statistically significant differences between groups, we set the second cutoff point to 1.35 times the first cutoff point. After obtaining the two cutoff points, we manually divided the data into three groups.

### Statistical analysis

For continuous variables with a normal distribution, Student’s *t*-test and one-way ANOVA are used, and the results are presented as mean ± standard deviation (SD). For skewed or non-normally distributed continuous variables, the Kruskal-Wallis rank-sum test is used for comparison, and the results are presented as median with interquartile range (IQRs). Fisher’s exact test or chi-square test is used for analyzing categorical variables, and the results are reported as numbers and percentages. We conducted a sensitivity analysis comparing the inclusion cohort with the exclusion cohort, which was defined by missing triglyceride, glucose, and BMI data. The baseline characteristics and crude outcomes between the inclusion and exclusion cohorts are presented in Supplementary Table 2. Kaplan–Meier curves were used to visualize the incidence of endpoints in survival analysis. Univariate and multivariate cox proportional hazard models were employed to determine the impact of TyG-BMI levels on the mortality in HS patients. The multivariate models included variables related to clinical outcomes and prognosis: Model 1: unadjusted; Model 2: adjusted for age, gender, and ethnicity; Model 3: based on the results of the univariate Cox regression analysis, adjusted for age, gender, SOFA score, hypertension, arrhythmias, liver failure, urea, white blood cells, hemoglobin, chloride, vasopressors, oxygen delivery, antiplatelet, anticoagulants, and lipid-lowering drugs. The results are reported as adjusted hazard ratios (aHRs) with 95% confidence intervals. Before including covariates in the multivariate model, we assessed multicollinearity using the variance inflation factor and reported the diagnostic results. Subgroup analyses were performed to determine the robustness of the result, with the results visually described through a forest plot. Restricted cubic spline (RCS) curve analysis was performed to investigate the dose-effect relationship between TyG-BMI and mortality in HS patients. The receiver operating characteristic curve was used to evaluate the predictive efficacy of TyG-BMI.

Statistical analyses were performed using STATA 14.0, R software (version 4.4.2), and SPSS 25.0 (IBM SPSS Statistics, Armonk, NY, United States). A two-tailed *p*-value < 0.05 was considered statistically significant.

## Results

### Baseline characteristics

As shown in Table 1, this study included 413 critically ill HS patients, with a median age of 66.77 (21.21), and 58.8% were male. Participants were divided into low (*n* = 80), intermediate (*n* = 186), and high (*n* = 147) TyG-BMI groups. There were significant differences between the three groups in terms of age, SOFA score, diabetes, sepsis, ventilator-associated pneumonia, white blood cells, creatinine, urea, international normalized ratio, oxygen delivery, vasopressor use, and anticoagulant medication. The high TyG-BMI group had a significantly lower median age (*p* = 0.001) and more severe condition which has indicated by SOFA scores (*p* = 0.002). The prevalence of comorbidities, including diabetes (*p* < 0.05), sepsis (*p* = 0.009), and ventilator-associated pneumonia (*p* = 0.001), was higher in the high TyG-BMI group. In laboratory indicators, the high TyG-BMI group had higher levels of white blood cells (*p* = 0.019), creatinine (*p* < 0.001), urea (*p* = 0.036), and international normalized ratio (*p* = 0.002). The low TyG-BMI group had a lower oxygen delivery rate (*p* = 0.025) and anticoagulant usage rate (*p* = 0.002). The intermediate TyG-BMI group had a lower vasopressor usage rate (*p* = 0.014). Regarding clinical outcomes, the high TyG-BMI group had a longer ICU stay (*p* = 0.001) and longer hospital stay (*p* = 0.008), while the intermediate TyG-BMI group had lower 30-day mortality (*p* = 0.025) and ICU mortality (*p* = 0.025).

**Table 1:** Baseline characteristics.

Variable	Overall	Low TyG-BMI (<201.6)	Middle TyG-BMI (201.7–271.9)	High TyG-BMI (>272.0)	*P* value
	413	80	186	147	
TyG-BMI	249.41 (90.18)	177.62 (31.60)	237.24 (34.07)	328.01 (86.79)	<0.001
Demographics					
Age, years	66.77 (21.21)	68.56 (21.24)	68.71 (21.78)	62.29 (18.96)	0.001
Men, *n* (%)	243 (58.8%)	38 (47.5%)	117 (62.9%)	88 (59.9%)	0.061
Body mass index, kg/cm^2^	27.81 (8.17)	20.71 (3.49)	26.56 (3.48)	34.65 (8.90)	<0.001
Race/ethnicity, *n* (%)					0.433
White	162 (39.2%)	35 (43.8%)	69 (37.1%)	58 (39.5%)	
Black	45 (10.9%)	7 (8.8%)	21 (11.3%)	17 (11.6%)	
Asian	13 (3.1%)	3 (3.8%)	9 (4.8%)	1 (0.7%)	
Other or unknown	193 (46.7%)	35 (43.8%)	87 (46.8%)	71 (48.3%)	
Clinical severity					
GCS	15 (3)	15 (3)	14 (3)	15 (2)	0.395
SOFA	3 (3)	3 (4)	3 (3)	4 (5)	0.002
APS III	39 (23)	38 (21)	37 (21)	40 (27)	0.075
Comorbidities					
Hypertension, *n* (%)	234 (56.7%)	45 (56.3%)	107 (57.5%)	82 (55.8%)	0.947
Diabetes mellitus, *n* (%)	123 (29.8%)	10 (12.5%)	47 (25.3%)	66 (44.9%)	<0.05
COPD, *n* (%)	22 (5.3%)	4 (5.0%)	9 (4.8%)	9 (6.1%)	0.865
Arrhythmias, *n* (%)	154 (37.3%)	24 (30.0%)	71 (38.2%)	59 (40.1%)	0.303
CAD, *n* (%)	55 (13.3%)	8 (10.0%)	25 (13.4%)	22 (15.0%)	0.574
Sepsis, *n* (%)	18 (4.4%)	0 (0.0%)	6 (3.2%)	12 (8.2%)	0.009
Liver disease, *n* (%)	36 (8.7%)	7 (8.8%)	14 (7.5%)	15 (10.2%)	0.691
Hyperlipemia, *n* (%)	3 (0.7%)	1 (1.3%)	2 (0.6%)	0	0.429
Ventilator-associated pneumonia, *n* (%)	67 (16.2%)	7 (8.8%)	23 (12.4%)	37 (25.2%)	0.001
Charlson comorbidity index, *n* (%)	6.0 (4.0)	6.0 (4.0)	6.0 (4.0)	5.0 (3.0)	0.234
Laboratory parameters					
Triglyceride, mg/dL	116.0 (96.0)	79.0 (47.0)	110.0 (76.0)	149.0 (124.0)	<0.001
Glucose, mg/dL	126.0 (58.0)	110.5 (39.0)	121.0 (54.0)	146.0 (72.0)	<0.001
RBC, K/uL	4.12 (0.86)	4.04 (0.86)	4.12 (0.82)	4.22 (0.85)	0.091
WBC, m/uL	11.2 (5.9)	9.9 (6.8)	10.9 (5.5)	12.2 (5.9)	0.019
Platelet, K/uL	206.0 (95.5)	208.0 (104.0)	193.0 (97.0)	219.0 (91.0)	0.076
Hemoglobin, g/dl	12.4 (2.9)	12.05 (3.1)	12.3 (3.0)	12.6 (2.7)	0.298
Creatinine, mg/dl	0.9 (0.5)	0.8 (0.4)	0.9 (0.4)	1.0 (0.5)	<0.001
Chloride, mEq/L	104.0 (6.0)	104.0 (7.0)	104.0 (6.0)	103.0 (7.0)	0.383
Bicarbonate, mEq/L	22.0 (4.0)	22.0 (4.0)	22.0 (3.0)	22.0 (3.0)	0.500
Urea, mg/dl	16.0 (12.0)	13.0 (10.0)	16.0 (11.0)	18.0 (13.0)	0.036
Prothrombin time, s	12.9 (2.1)	12.6 (2.4)	13.0 (2.2)	13.1 (1.8)	0.129
International normalized ratio	1.2 (0.2)	1.2 (0.2)	1.2 (0.2)	1.2 (0.3)	0.002
Treatment					
Vasopressors, *n* (%)	94 (22.8%)	22 (27.5%)	30 (16.1%)	42 (28.6%)	0.014
Oxygen delivery, *n* (%)	270 (65.4%)	42 (52.5%)	126 (67.7%)	102 (69.4%)	0.025
Antiplatelet	124 (30.0%)	18 (22.5%)	60 (32.3%)	46 (31.3%)	0.258
Anticoagulation	366 (88.6%)	62 (77.5%)	171 (91.9%)	133 (90.5%)	0.002
Lipid-lowering	115 (27.8%)	21 (26.3%)	53 (28.5%)	41 (27.9%)	0.932
Clinical outcomes					
LOS ICU, day	9.77 (12.99)	7.31 (10.45)	8.99 (10.90)	12.55 (15.16)	0.001
LOS Hospital, day	15.50 (18.99)	11.09 (17.35)	14.94 (17.91)	18.25 (21.75)	0.008
All-cause mortality					
In-hospital mortality, *n* (%)	110 (26.6%)	23 (28.7%)	41 (22.0%)	46 (41.8%)	0.148
ICU mortality, *n* (%)	98 (23.7%)	17 (21.3%)	35 (18.8%)	46 (31.3%)	0.025
30-day mortality, *n* (%)	134 (32.4%)	35 (43.8%)	50 (26.9%)	49 (33.3%)	0.025
90-day mortality, *n* (%)	160 (38.7%)	38 (47.5%)	62 (33.3%)	60 (40.8%)	0.076
1-year mortality, *n* (%)	185 (44.8%)	43 (53.8%)	74 (39.8%)	68 (46.3%)	0.100

GCS, Glasgow coma scale; SOFA, sequential organ failure assessment; APSIII, acute physiology scores III; COPD, chronic pulmonary disease; CAD, coronary atherosclerotic heart disease; RBC, red blood cell; WBC, white blood cell. LOS, length of stay.

### Associations between TyG-BMI and clinical outcomes

The results of the univariate Cox regression analysis and the Multicollinearity Diagnosis are shown in Supplementary Tables 3, 4. Multivariable Cox regression analysis was conducted to assess the effect of TyG-BMI on the mortality risk at different time points in patients with HS, which is shown in Table 2 and Supplementary Table 5. The results revealed that in both the unadjusted model (Model 1) and the model adjusted for age, gender, and race (Model 2), the low TyG-BMI group (Tertile 1) had a significantly higher risk of mortality at 30 days [Model 1: aHR = 1.863, 95% CI: 1.209–2.869, *p* = 0.005; Model 2: aHR = 1.842, 95% CI: 1.190–2.852, *p* = 0.006], 90 days, and 1 year compared to the intermediate TyG-BMI group (Tertile 2), whereas there was no significant difference between the high TyG-BMI group (Tertile 3) and the reference group (Tertile 2) (all *p* > 0.05). In Model 3, the low TyG-BMI group (Tertile 1) had a significantly higher risk of mortality at 30 days (HR = 1.836, 95% CI: 1.158–2.911, *p* = 0.010), 90 days (HR = 1.694, 95% CI: 1.104–2.601, *p* = 0.016), and 1 year (HR = 1.642, 95% CI: 1.107–2.437, *p* = 0.014) compared to the reference group. The high TyG-BMI group (Tertile 3) also had a significantly higher risk of mortality at 30 days (HR = 1.584, 95% CI: 1.024–2.453, *p* = 0.039), 90 days (HR = 1.571, 95% CI: 1.062–2.323, *p* = 0.024), and 1 year (HR = 1.484, 95% CI: 1.038–2.121, *p* = 0.030) compared to the reference group. These findings suggest that both the low and high TyG-BMI groups are associated with a higher mortality risk, while intermediate TyG-BMI levels have a protective effect for HS patients. Additionally, the trend test (P for trend) results across all models were not statistically significant (all *p* > 0.05), indicating that there may not be a linear dose–response relationship between TyG-BMI and mortality risk, but rather a U-shaped association.

**Table 2:** Cox proportional hazard ratios for all-cause mortality at 30-day, 90-day, and 1-year.

	Model 1		Model 2		Model 3	
	HR, 95%CI	*P* value	HR, 95%CI	*P* value	HR, 95%CI	*P* value
30-day mortality						
Tertile 1	1.863 (1.209–2.869)	0.005	1.842 (1.190–2.852)	0.006	1.836 (1.158–2.911)	0.010
Tertile 2	Ref		Ref		Ref	
Tertile 3	1.278 (0.862–1.895)	0.222	1.341 (0.899–1.998)	0.150	1.584 (1.024–2.453)	0.039
P for trend	0.857 (0.677–1.09)	0.202	0.884 (0.695–1.12)	0.315	0.954 (0.733–1.241)	0.726
90-day mortality						
Tertile 1	1.657 (1.106–2.482)	0.014	1.624 (1.079–2.443)	0.020	1.694 (1.104–2.601)	0.016
Tertile 2	Ref		Ref		Ref	
Tertile 3	1.278 (0.896–1.822)	0.176	1.351 (0.943–1.936)	0.102	1.571 (1.062–2.323)	0.024
P for trend	0.915 (0.735–1.14)	0.423	0.951 (0.762–1.19)	0.660	1.002 (0.787–1.277)	0.984
1-year mortality						
Tertile 1	1.591 (1.093–2.318)	0.015	1.549 (1.059–2.266)	0.024	1.642 (1.107–2.437)	0.014
Tertile 2	Ref		Ref		Ref	
Tertile 3	1.223 (0.880–1.701)	0.230	1.304 (0.934–1.822)	0.119	1.484 (1.038–2.121)	0.030
P for trend	0.91 (0.742–1.11)	0.361	0.953 (0.775–1.17)	0.650	0.989 (0.792–1.235)	0.920

HR, hazard ratio; CI, confidence interval; Model 1: unadjusted. Model 2: adjusted age, gender, and ethnicity. Model 3: adjusted for age, gender, SOFA score, hypertension, arrhythmias, liver failure, urea, white blood cells, hemoglobin, chloride, vasopressors, oxygen delivery, antiplatelet, anticoagulants, and lipid-lowering drugs.

**Figure 2:**
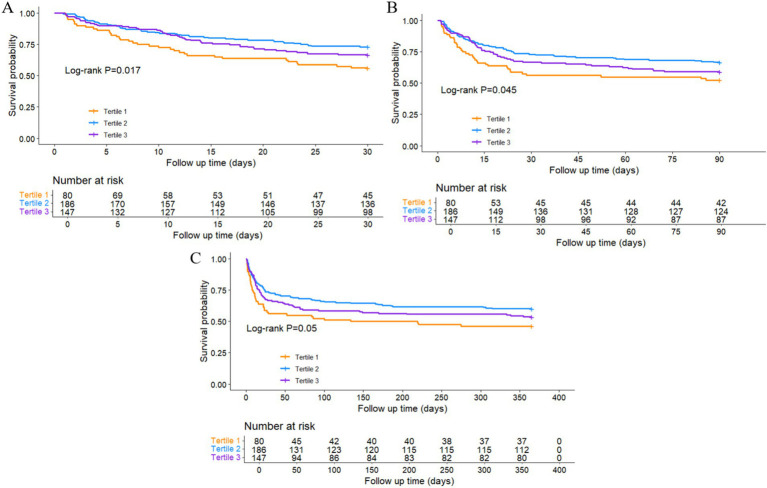
Kaplan–Meier survival analysis curves for **(A)** 30-day, **(B)** 90-day, and **(C)** 1-year all-cause mortality.

The Kaplan–Meier curve was used to compare the mortality outcomes among different TyG-BMI subgroups in HS patients. The analysis revealed significant differences in survival curves between the groups at 30-day (Log-rank *p* = 0.017; Figure 2A) and 90-day (Log-rank *p* = 0.045; Figure 2B), and a marginally significant difference at 1-year (Log-rank *p* = 0.050; Figure 2C). Patients in the lowest TyG-BMI group had the lowest survival rate, while those in the intermediate TyG-BMI group had the highest survival rate. The difference among the three groups in terms of ICU and in-hospital mortality did not reach statistical significance, with log-rank *p* values of 0.42 and 0.22, respectively (Supplementary Figure 1).

### Relationship between the TyG-BMI and mortality risk

**Figure 3:**
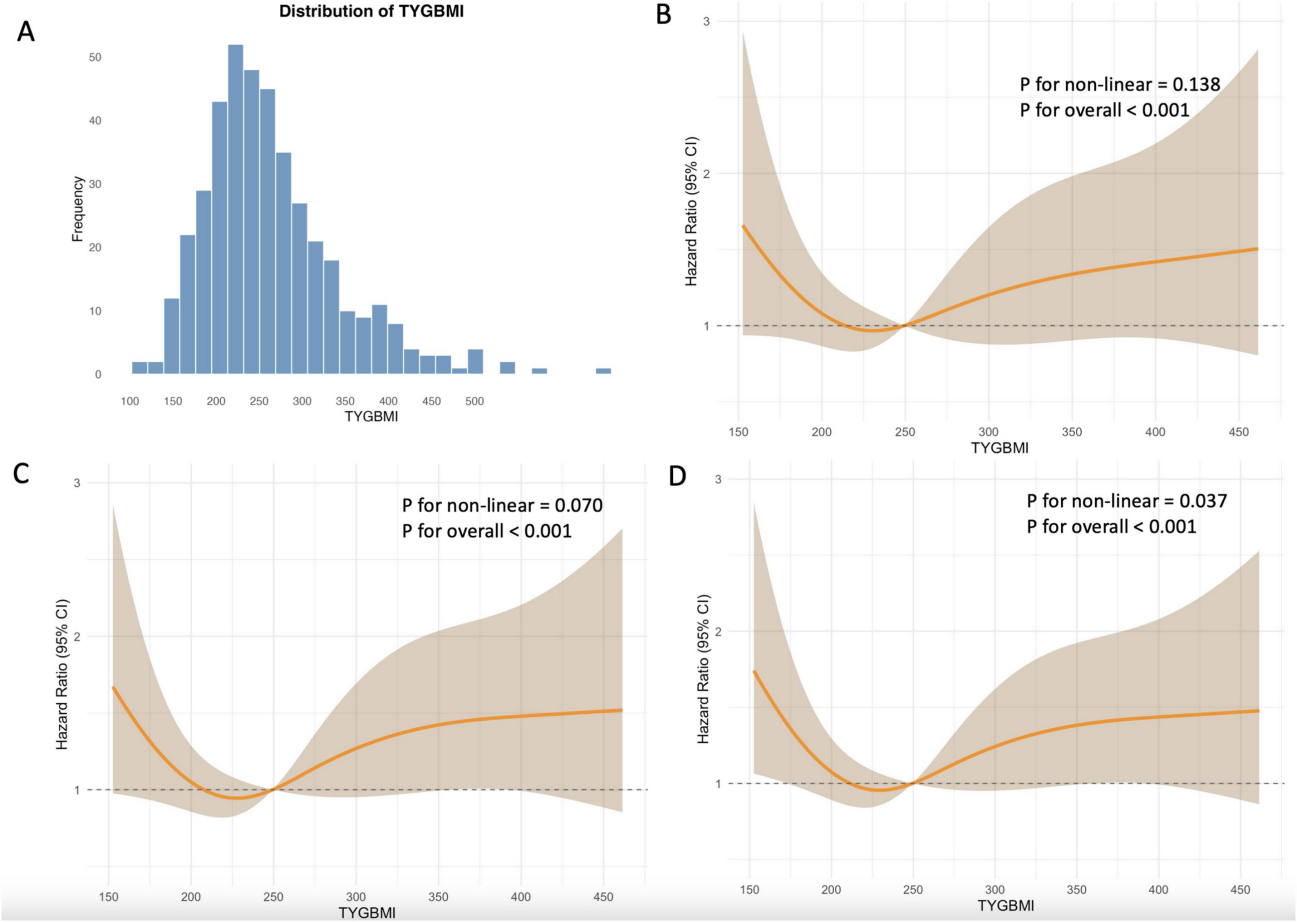
The **(A)** distribution of TyG-BMI and the restricted cubic spline curve for **(B)** 30-day, **(C)** 90-day, and **(D)** 1-year all-cause mortality.

Figure 3A shows the distribution of TyG-BMI. Across the 30-day (Figure 3B), 90-day (Figure 3C), or 1-year point (Figure 3D), all three RCS curves display a similarly U-shaped pattern. At lower TyG-BMI values, an increase in the index is associated with a reduction in mortality risk. However, once a certain threshold is reached, the mortality risk increases as TyG-BMI continues to rise beyond this threshold. The RCS curves for ICU and in-hospital mortality risk are shown in Supplementary Figure 2.

The predictive value of clinical TyG-BMI for mortality was assessed using receiver operating characteristic curves (Supplementary Figure 3). However, the area under the curve (AUC) values showed limited predictive ability (30-day mortality AUC: 0.529; 90-day mortality AUC: 0.513; 1-year mortality AUC: 0.523).

### Subgroup analysis

**Figure 4:**
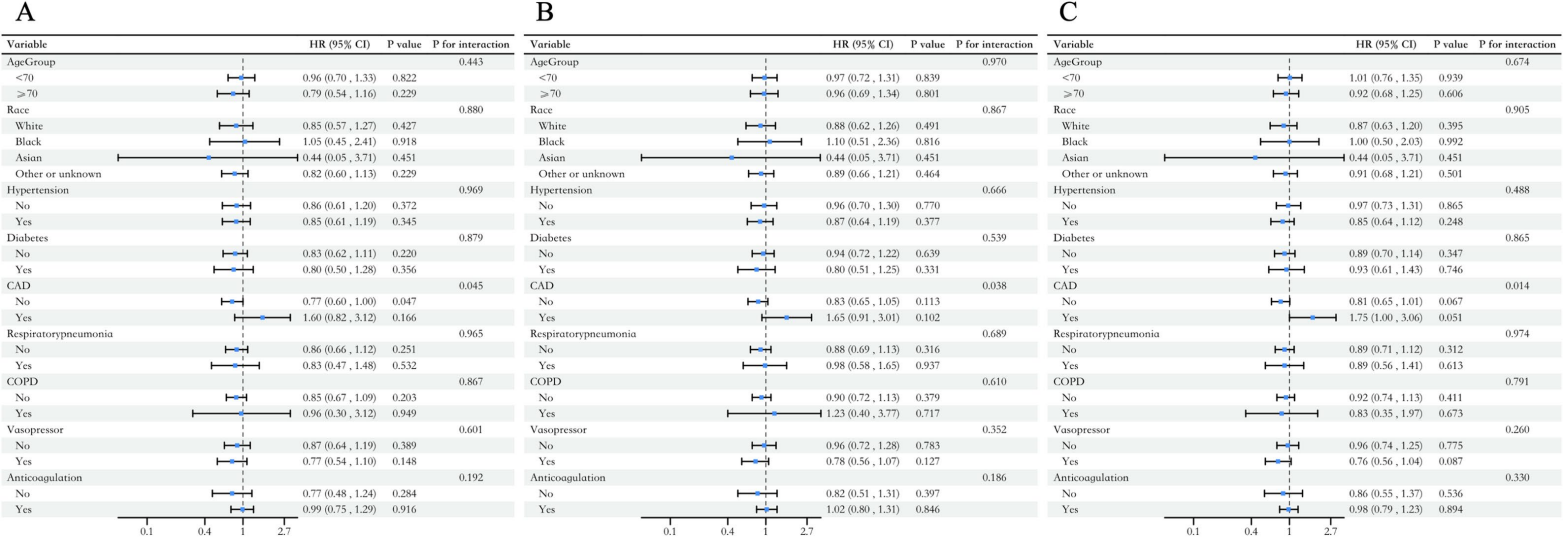
Forest plots of hazard ratios for **(A)** 30-day, **(B)** 90-day, **(C)** 1-year all-cause mortality across different subgroups.

To further assess the relationship between TyG-BMI and mortality, we conducted subgroup analyses to evaluate the associations between age (<70 years and ≥70 years), race, hypertension, diabetes, CAD, ventilator-associated pneumonia, COPD, use of vasopressors, and anticoagulants with all-cause mortality at 30-day (Figure 4A), 90-day (Figure 4B), and 1-year (Figure 4C) in HS patients. The subgroup analysis showed that TyG-BMI was significantly associated with a lower risk of mortality at 30-day in HS patients without coronary artery disease (HR 0.77, 95% CI: 0.60–1.00, *p* = 0.047), and no other subgroup factors significantly influenced the relationship between TyG-BMI and all-cause mortality in HS patients. Interaction tests revealed a significant interaction between coronary artery disease and the 30-day, 90-day, and 1-year mortality risks in HS patients (all interaction *p*-values < 0.05). For ICU and in-hospital mortality (Supplementary Figure 4), no subgroup factors had a significant effect, and only the use of anticoagulants showed an interaction with in-hospital mortality (p for interaction = 0.007).

## Discussion

This study is the first to explore the relationship between TyG-BMI and mortality risk in HS patients using the MIMIC database. It revealed a U-shaped relationship between TyG-BMI and 30-day mortality rates, demonstrating that both low and high levels of TyG-BMI levels are associated with an increased risk of mortality in the United States patient population. Notably, compared to the intermediate TyG-BMI group, the 30-day mortality risk in the low TyG-BMI group was increased by 83.6% (HR = 1.836, *p* = 0.010), while the 30-day mortality risk in the high TyG-BMI group increased by 58.4% (HR = 1.584, *p* = 0.039). This pattern remained evident in the 90-day and 1-year mortality analyses. These findings highlighting its potential as a feasible biomarker for predicting mortality risk in HS patients.

The TyG index, a reliable surrogate marker for IR, has become an important predictive tool for stroke risk management in recent years (11–13). Multicenter cohort studies have demonstrated that, compared to stroke patients with lower TyG indices, those with higher TyG indices exhibit significantly greater clinical risks. Specifically, the risk of recurrent stroke increases by 50% (OR = 1.50, 95%CI 1.19–1.89), and the risk of all-cause mortality increases by 40% (OR = 1.40, 95%CI 1.14–1.71) (14). The TyG index has also been linked to an increased incidence of carotid plaque (15).

Obesity, a core component of metabolic syndrome, is closely linked to classic stroke risk factors such as hypertension, diabetes, and dyslipidemia. However, recent studies have revealed a complex relationship between obesity and hemorrhagic stroke prognosis. A study involving 234,863 Korean men demonstrated a J-shaped association between BMI and HS (16). Since obesity leads to hypertension, diabetes, and hypercholesterolemia, these variables may contribute to the increased stroke risk associated with obesity. Interestingly, clinical observations of ICH patients further revealed that in-hospital mortality rates were significantly reduced by 31% (OR = 0.69; 95%CI = 0.62–0.76; *p* < 0.001) in the obesity group (BMI ≥ 30 kg/m^2^) and by 15% (OR = 0.85; 95%CI = 0.74–0.97; *p* = 0.02) in the morbid obesity group (BMI > 40 kg/m^2^), compared to normal weight patients (17). Notably, prospective studies have shown that underweight patients (BMI < 18.5 kg/m^2^) exhibit a significantly higher risk (41%) of severe functional impairment (Modified Rankin Scale 5–6) after ICH, compared to those with normal weight (OR: 1.41 [1.01–1.99]). This could be due to decreased tissue repair capacity and impaired immune response caused by malnutrition (18).

TyG-BMI, a composite metabolic index that combines fasting blood glucose, triglycerides, and BMI, has recently gained widespread recognition as a sensitive tool for evaluating IR and cardiovascular metabolic risks (19–21). By integrating both glucose-lipid metabolism abnormalities and obesity, TyG-BMI is considered to provide a more comprehensive reflection of the pathophysiological processes associated with IR compared to individual indices. A moderate TyG-BMI may suggest a balance in energy reserves, potentially placing the body in an optimal state between pro-inflammatory and anti-inflammatory activities. This balance may better support the body’s response to insulin resistance and neuroinflammatory responses during acute hemorrhage, facilitate tissue repair and stress demands (22), and reduce endothelial dysfunction caused by impaired nitric oxide (NO) synthesis due to high IR. It may also reduce the release of adipose tissue inflammatory factors, thereby mitigating systemic inflammation (23). The persistent elevation of TyG-BMI can trigger the continuous release of inflammatory mediators, such as CRP and various cytokines. These inflammatory factors may adversely affect the structural integrity and functional capacity of the vascular endothelium (9). Low TyG-BMI may reflect severe energy deficiency (such as malnutrition or cachexia) or excessive depletion. In malnutrition, energy reserves are low, accompanied by immune suppression and abnormal inflammatory responses, limiting the body’s ability to respond to acute hemorrhage.

We employed RCS to explore the dose–response relationship between IR surrogate indices and HS. Our findings partially align with previous studies, while also revealing important distinctions. Unlike the L-shaped correlation reported by Huang et al. in stroke populations (8), we observed that both low and high TyG-BMI were associated with an increased risk of HS mortality. The higher mortality risk in HS patients with high TyG-BMI may be related to the following reasons: high TyG-BMI exacerbates IR, leading to endothelial dysfunction (reduced nitric oxide synthesis and increased endothelin) (24), which promotes vasospasm and re-rupture. High TyG-BMI also triggers systemic inflammation (increased TNF-*α*, IL-6) (23), exacerbating perivascular neuroinflammation and secondary brain injury (25). Notably, a study based on the eICU database found high TyG-BMI was associated with an increased risk of mortality in ischemic stroke patients (9), aligns with our finding that high TyG-BMI is associated with increased HS mortality.

The biological plausibility of our study’s findings may be explained by the following complementary mechanisms. First, the protective effect observed at intermediate TyG-BMI levels may reflect an optimal balance of energy reserves and metabolic flexibility, thereby providing protection. As a composite measure of glucose-lipid metabolism and nutritional status, the ideal TyG-BMI level likely balances the utilization of energy substrates with the regulation of inflammation. Intermediate metabolic activity helps the body cope with acute stress responses while avoiding metabolic imbalances caused by excessive obesity or malnutrition. This balance may mitigate or prevent secondary injury triggered by metabolic disturbances and excessive inflammatory responses. Second, lower TyG-BMI levels may indicate severe malnutrition or cachexia, conditions that impair immune function, decrease tissue repair capacity, and increase the risk of infection. In the context of hemorrhagic stroke, these factors can significantly affect patient survival and prognosis.

Our study does have several limitations. First, as a retrospective, single-center analysis based on observational data from the MIMIC-IV database. Although we adjusted for various variables and conducted subgroup analyses, we cannot completely rule out the potential influence of unmeasured confounders on the outcomes. Sensitivity analysis indicated that the exclusion of patients due to missing triglyceride, glucose, and BMI data resulted in some selection bias. We observed that patients in the excluded cohort appeared less acutely ill and had a lower documented burden of relevant comorbidities (especially hypertension, diabetes, CAD). Clinicians may have deemed comprehensive metabolic profiling less immediately necessary for diagnosis or management in these patients. Second, the relatively small sample size limits the generalizability of our findings, and larger cohort studies are needed to further validate our conclusions. Third, the categorization of the TyG-BMI index into three groups based on statistically optimized cutoffs introduces methodological constraints. While this approach aimed to maximize survival differences and ensure comparability between groups, the thresholds used lack established clinical relevance. Future research should focus the prospective validation of standardized TyG-BMI values across diverse ethnic populations, and the integration of inflammatory biomarkers a in mechanistic studies.

## Conclusion

This study found that the TyG-BMI index is a potential predictor of 30-day all-cause mortality in patients with HS. There is a similarly U-shaped relationship between TyG-BMI levels and all-cause mortality in these patients. The TyG-BMI index can serve as an effective marker for stratifying and managing HS patients in the ICU.

## Data Availability

Publicly available datasets were analyzed in this study. This data can be found here: https://physionet.org/content/mimiciv/3.0/.
