# Supplementary material for: Associations between triglyceride glucose-body mass index and 30-day mortality in patients with hemorrhagic stroke: analysis of the MIMIC-IV database
Source: Front Neurol. 2025 Aug 8;16:1602822. doi: 10.3389/fneur.2025.1602822 (PMC12370647; doi:10.3389/fneur.2025.1602822)
Supplement: Supplementary file 1 [file Table_1.docx]

**Supplemental Table 1.** Missing values of included individuals.

| **Variable** | **Missing** |
| --- | --- |
| **Demographics** |  |
| Age | 0 |
| Gender | 0 |
| Race | 0 |
| Height | 0 |
| Weight | 0 |
| **Clinical severity** |  |
| GCS | 0 |
| SOFA | 0 |
| APS III | 0 |
| **Comorbidities** |  |
| Hypertension | 0 |
| Diabetes mellitus | 0 |
| COPD | 0 |
| Arrhythmias | 0 |
| CAD | 0 |
| Sepsis | 0 |
| Liver disease | 0 |
| Hyperlipemia | 0 |
| Respiratory pneumonia | 0 |
| Charlson comorbidity index | 0 |
| **Laboratory parameters** |  |
| RBC | 2 (0.5%) |
| WBC | 3 (7.3%) |
| Platelet | 21 (5.1%) |
| Hemoglobin | 3 (7.3%) |
| Albumin | 132 (32.0%) |
| CRP | 301 (72.9%) |
| Lymphocytes | 158 (38.3%) |
| Neutrophils | 158 (38.3%) |
| Sodium | 121 (29.3%) |
| Glucose | 0 |
| Potassium | 108 (26.2%) |
| Creatinine | 4 (1.0%) |
| Lactate | 144 (34.9%) |
| Chloride | 9 (2.2%) |
| Cholesterol | 88 (21.3%) |
| Bicarbonate | 13 (3.1%) |
| Urea | 0 |
| Prothrombin time | 0 |
| International normalized ratio | 0 |
| **Treatment** |  |
| Vasopressors | 0 |
| Oxygen delivery | 0 |
| Antiplatelet | 0 |
| Anticoagulation | 0 |
| Lipid-lowering | 0 |

Abbreviation: GCS, Glasgow coma scale; SOFA, sequential organ failure assessment; APSIII, acute physiology scores III; COPD, chronic pulmonary disease; CAD, coronary atherosclerotic heart disease; RBC, red blood cell; WBC, white blood cell.

**Supplemental Table 2.** Baseline characteristics and crude outcomes between the inclusion and exclusion cohorts.

| **Variable** | **Inclusion cohort(n=413)** | **Exclusion cohort (n=3315)** | **P value** | |  |
| --- | --- | --- | --- | --- | --- |
| **Demographics** | | | |  | |
| Age, years | 66.77 (21.21) | 67.79 (23.85) | 0.041 | |  |
| Men, n (%) | 243 (58.8%) | 1645 (49.5%) | <0.001 | |  |
| Race/ethnicity, n (%) <0.001 | | | |  | |
| White | 162 (39.2%) | 2047 (61.6%) |  | |  |
| Black | 45 (10.9%) | 252 (7.6%) |  |  |  |
| Asian | 13 (3.1%) | 129 (3.9%) |  |  |  |
| Other or unknown | 193 (46.7%) | 896 (27.0%) |  |  |  |
| **Clinical severity** | | | |  | |
| GCS | 15 (3) | 14 (3) | <0.001 | |  |
| SOFA | 3 (3) | 3 (3) | <0.001 | |  |
| APS III | 39 (23) | 33 (21) | <0.001 | |  |
| **Comorbidities** | | | |  | |
| Hypertension, n (%) | 234 (56.7%) | 973 (29.5%) | <0.001 | |  |
| Diabetes mellitus, n (%) | 123 (29.8%) | 696 (20.9%) | < 0.001 | |  |
| COPD, n (%) | 22 (5.3%) | 116 (3.5%) | 0.062 | |  |
| Arrhythmias, n (%) | 154 (37.3%) | 528 (15.9%) | <0.001 | |  |
| CAD, n (%) | 55 (13.3%) | 211 (6.3%) | <0.001 | |  |
| Sepsis, n (%) | 18 (4.4%) | 77 (2.3%) | 0.013 | |  |
| Liver disease, n (%) | 36 (8.7%) | 43 (1.3%) | <0.001 | |  |
| Hyperlipemia, n (%) | 3 (0.7%) | 467 (14.0%) | <0.001 | |  |
| Ventilator-associated pneumonia, n (%) | 67 (16.2%) | 163 (4.9%) | <0.001 | |  |
| Charlson comorbidity index, n (%) | 6.0 (4.0) | 5.0 (4.0) | <0.001 | |  |
| **Laboratory parameters** | | | |  | |
| RBC, K/uL | 4.12 (0.86) | 4.09 (0.86) | 0.432 | |  |
| WBC, m/uL | 11.2 (5.9) | 10.3 (5.4) | <0.001 | |  |
| Platelet, K/uL | 206.0 (95.5) | 212.0 (93.0) | 0.254 | |  |
| Hemoglobin, g/dl | 12.4 (2.9) | 12.3 (2.5) | 0.988 | |  |
| Creatinine, mg/dl | 0.9 (0.5) | 0.8 (0.4) | <0.001 | |  |
| Chloride, mEq/L | 104.0 (6.0) | 104.0 (6.0) | 0.443 | |  |
| Bicarbonate, mEq/L | 22.0 (4.0) | 23.0 (4.0) | <0.001 | |  |
| Urea, mg/dl | 16.0 (12.0) | 15.0 (10.0) | 0.004 | |  |
| Prothrombin time, s | 12.9 (2.1) | 12.3 (2.2) | <0.001 | |  |
| International normalized ratio | 1.2 (0.2) | 1.1 (0.2) | <0.001 | |  |
| **Treatment** | | | |  | |
| Vasopressors, n (%) | 94 (22.8%) | 412 (12.4%) | <0.001 | |  |
| Oxygen delivery, n (%) | 270 (65.4%) | 2006 (60.3%) | 0.048 | |  |
| Antiplatelet | 124 (30.0%) | 574 (17.3%) | <0.001 | |  |
| Anticoagulation | 366 (88.6%) | 1972 (59.3%) | <0.001 | |  |
| Lipid-lowering | 115 (27.8%) | 482 (14.5%) | <0.001 | |  |
| **Clinical outcomes** |  |  |  | |  |
| LOS Hospital, day | 9.77 (12.99) | 3.14 (6.04) | <0.001 | |  |
| LOS Hospital, day | 15.50 (18.99) | 7.78 (10.82) | <0.001 | |  |
| **All-cause mortality** | | | |  | |
| In-hospital mortality, n (%) | 110 (26.6%) | 694 (20.9%) | 0.007 | |  |
| ICU mortality, n (%) | 98 (23.7%) | 554 (16.7%) | <0.001 | |  |
| 30-day mortality, n (%) | 134 (32.4%) | 879 (26.4%) | 0.010 | |  |
| 90-day mortality, n (%) | 160 (38.7%) | 1025 (30.8%) | 0.001 | |  |
| 1-year mortality, n (%) | 185 (44.8%) | 1225 (36.9%) | 0.002 | |  |

Abbreviation: GCS, Glasgow coma scale; SOFA, sequential organ failure assessment; APSIII, acute physiology scores III; COPD, chronic pulmonary disease; CAD, coronary atherosclerotic heart disease; RBC, red blood cell; WBC, white blood cell. LOS, length of stay.

**Supplemental Table 3**. Univariate Cox proportional hazard ratio for 30-day mortality.

| **Variable** | **HR, 95%CI** | **P value** |  |
| --- | --- | --- | --- |
| TyG-BMI | | | |
| Tertile 1 | Ref |  |  |
| Tertile 2 | 0.537 (0.348-0.827) | 0.005 |  |
| Tertile 3 | 0.686 (0.445-1.059) | 0.089 |  |
| Age | 1.010 (1.000-1.030) | 0.025 |  |
| BMI | 0.987 (0.964-1.010) | 0.300 |  |
| Gender | 0.752 (0.536-1.060) | 0.100 |  |
| White | Ref |  |  |
| Black | 1.106 (0.595-2.060) | 0.750 |  |
| Asian | 0.887 (0.275-2.860) | 0.840 |  |
| Other or unknown | 1.596 (1.097-2.320) | 0.015 |  |
| SOFA | 1.120 (1.070-1.160) | <0.001 |  |
| APSIII 1.020 (1.020-1.030) <0.001 | | | |
| GCS 0.980 (0.928-1.030) 0.460 | | | |
| OASIS 1.070 (1.050-1.090) <0.001 | | | |
| Hypertension | 0.739 (0.527-1.040) | 0.080 |  |
| Diabetes | 1.150 (0.799-1.650) | 0.450 |  |
| COPD | 0.674 (0.276-1.650) | 0.390 |  |
| Arrhythmias | 1.380 (0.984-1.950) | 0.062 |  |
| CAD | 1.250 (0.785-1.990) | 0.350 |  |
| Liver disease | 2.540 (1.620-3.980) | <0.001 |  |
| Sepsis | 1.150 (0.537-2.460) | 0.720 |  |
| Ventilator-associated pneumonia | 0.999 (0.637-1.56) | 1.000 |  |
| Charlson comorbidity index | 1.060 (0.996-1.130) | 0.068 |  |
| Creatinine | 1.16 (1.040-1.290) | 0.008 |  |
| Urea | 1.020 (1.010-1.020) | <0.001 |  |
| WBC | 1.010 (1.000-1.010) | 0.022 |  |
| RBC | 0.673 (0.547-0.828) | <0.001 |  |
| Hemoglobin | 0.866 (0.805-0.931) | <0.001 |  |
| Bicarbonate | 0.959 (0.906-1.010) | 0.150 |  |
| Chloride | 1.040 (1.000-1.070) | 0.032 |  |
| Platelet | 0.998 (0.996-1.000) | 0.110 |  |
| Prothrombin time 1.020 (0.996-1.040) 0.110 | | | |
| Vasopressor | 2.250 (1.580-3.190) | <0.001 |  |
| Oxygen delivery | 0.218 (0.154-0.309) | <0.001 |  |
| Anticoagulation | 0.293 (0.193-0.445) | <0.001 |  |
| Antiplatelet | 0.575 (0.381-0.868) | 0.009 |  |
| Lipid-lowering | 0.498 (0.320-0.774) | 0.002 |  |

**Supplemental Table 4.** Multicollinearity Diagnosis (Variance-Inflation Factor Results)

| **Variable** | **VIF** |
| --- | --- |
| Age | 2.41 |
| Gender | 1.29 |
| Race |  |
| White | Ref |
| Black | 1.30 |
| Asian | 1.10 |
| Other or unknown | 1.30 |
| GCS | 1.67 |
| SOFA | 3.19 |
| APSIII | 2.66 |
| Diabetes | 1.54 |
| CAD | 1.30 |
| COPD | 1.10 |
| Sepsis | 1.26 |
| Hyperlipemia | 1.09 |
| Respiratory pneumonia | 1.21 |
| Liver failure | 1.49 |
| Hypertension | 1.33 |
| Arrhythmias | 1.28 |
| Charlson comorbidity index | 2.90 |
| Urea | 2.54 |
| WBC | 1.06 |
| RBC | 6.13 |
| Creatinine | 2.49 |
| Hb | 6.32 |
| Chloride | 1.28 |
| Platelet | 1.43 |
| Bicarbonate | 1.26 |
| INR | 1.08 |
| Prothrombin time | 1.21 |
| Vasopressor | 1.37 |
| Oxygen delivery | 1.27 |
| Anticoagulation | 1.21 |
| Antiplatelet | 1.36 |
| Lipid lowering | 1.33 |

Abbreviation: VIF, variance-inflation factor; GCS, Glasgow coma scale; SOFA, sequential organ failure assessment; APSIII, acute physiology scores III; COPD, chronic pulmonary disease; CAD, coronary atherosclerotic heart disease; RBC, red blood cell; WBC, white blood cell

**Supplemental Table 5**. Cox proportional hazard ratios for all-cause mortality at ICU and in-hospital.

|  | **Model 1** | | **Model 2** | | **Model 3** | |
| --- | --- | --- | --- | --- | --- | --- |
|  | **HR, 95%CI** | **P value** | **HR, 95%CI** | **P value** | **HR, 95%CI** | **P value** |
| ICU mortality | | | | | | |
| Tertile 1 | 1.356 (0.758-2.425) | 0.305 | 1.317 (0.732-2.369) | 0.358 | 1.077 (0.574-2.021) | 0.818 |
| Tertile 2 | Ref |  | Ref |  | Ref |  |
| Tertile 3 | 1.305 (0.840-2.029) | 0.237 | 1.286 (0.821-2.014) | 0.272 | 1.632 (0.992-2.684) | 0.054 |
| P for trend | 1.045 (0.787-1.387) | 0.763 | 1.045 (0.785-1.390) | 0.764 | 1.297 (0.948-1.775) | 0.104 |
| In-hospital mortality | | | | | | |
| Tertile 1 | 1.536 (0.921-2.563) | 0.100 | 1.590 (0.947-2.670) | 0.079 | 1.145 (0.661-1.984) | 0.629 |
| Tertile 2 | Ref |  | Ref |  | Ref |  |
| Tertile 3 | 1.285 (0.843-1.959) | 0.244 | 1.306 (0.805-2.005) | 0.223 | 1.407 (0.874-2.263) | 0.160 |
| P for trend | 0.965 (0.742-1.256) | 0.791 | 1.045 (0.735-1.253) | 0.760 | 1.139 (0.851-1.523) | 0.381 |

HR: hazard ratio; CI: confidence interval; Model 1: unadjusted. Model 2: adjusted age, gender, and ethnicity. Model 3: adjusted for age, gender, SOFA score, hypertension, arrhythmias, liver failure, urea, white blood cells, hemoglobin, chloride, vasopressors, oxygen delivery, antiplatelet, anticoagulants, and lipid-lowering drugs.

**Supplemental Figure 1.** Kaplan-Meier survival analysis curves for **(A)** ICU and **(B)** in-hospital mortality.


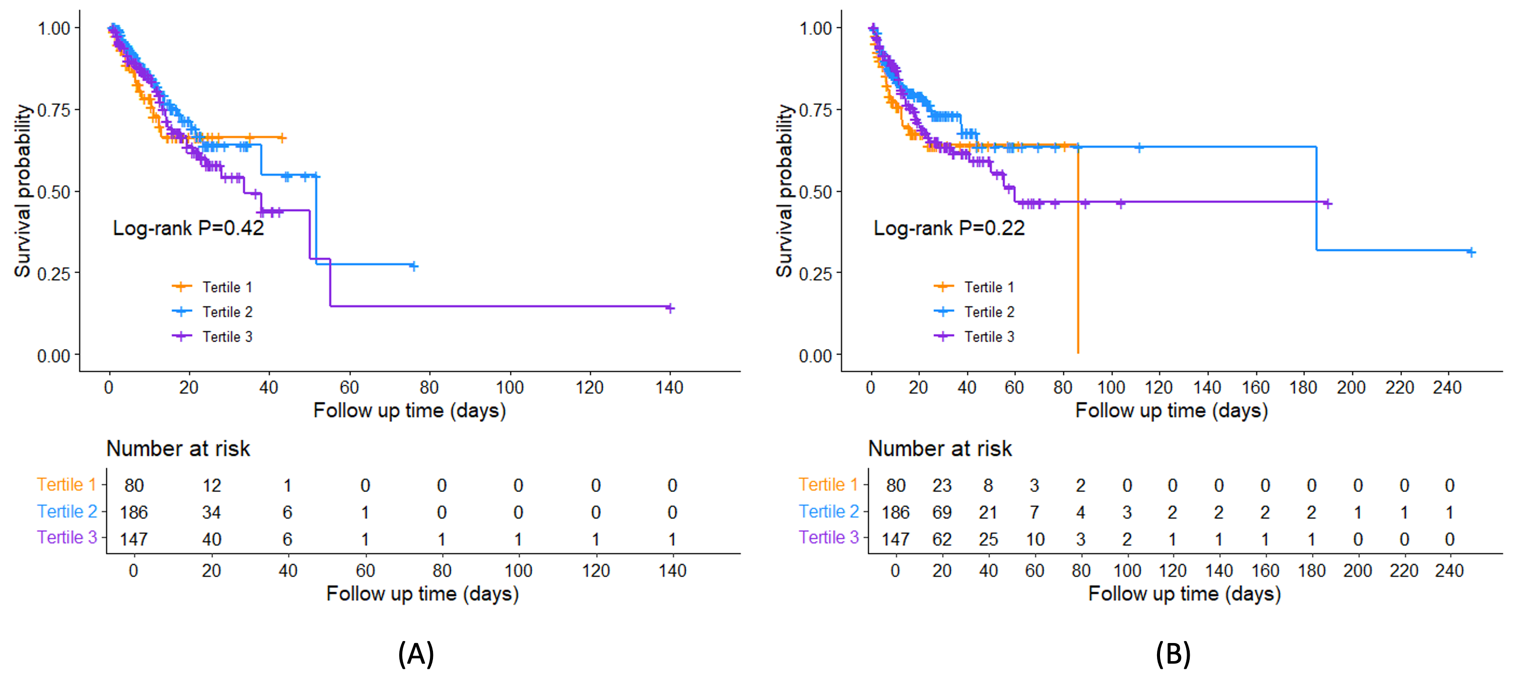


ICU: intensive care unit.

**Supplemental Figure 2.** Restricted cubic spline curve for **(A)** ICU and **(B)** in-hospital mortality.


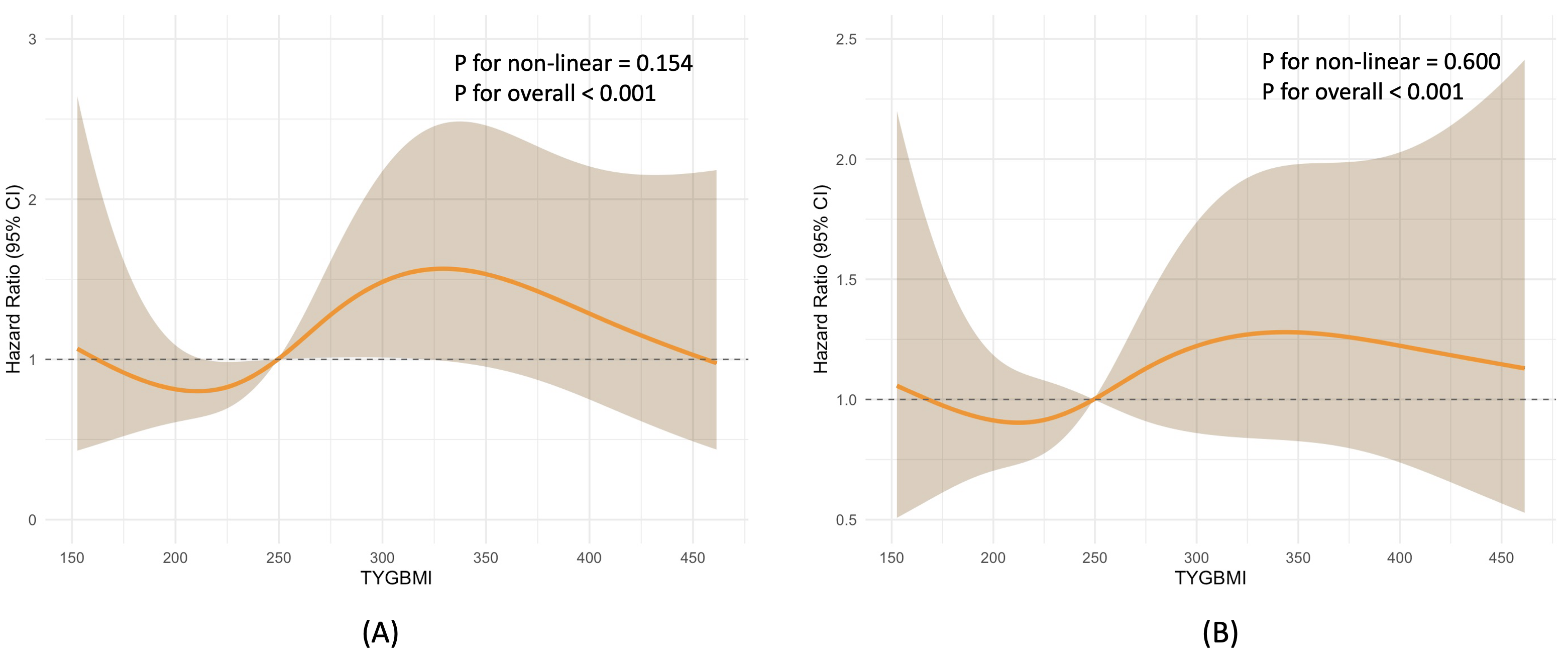


CI, confidence interval; TyG-BMI, triglyceride glucose-body mass index; ICU: intensive care unit.

**Supplemental Figure 3.** Receiver operating characteristic curve for **(A)** 30-day **(B)** 90-day and **(C)** 1-year mortality.


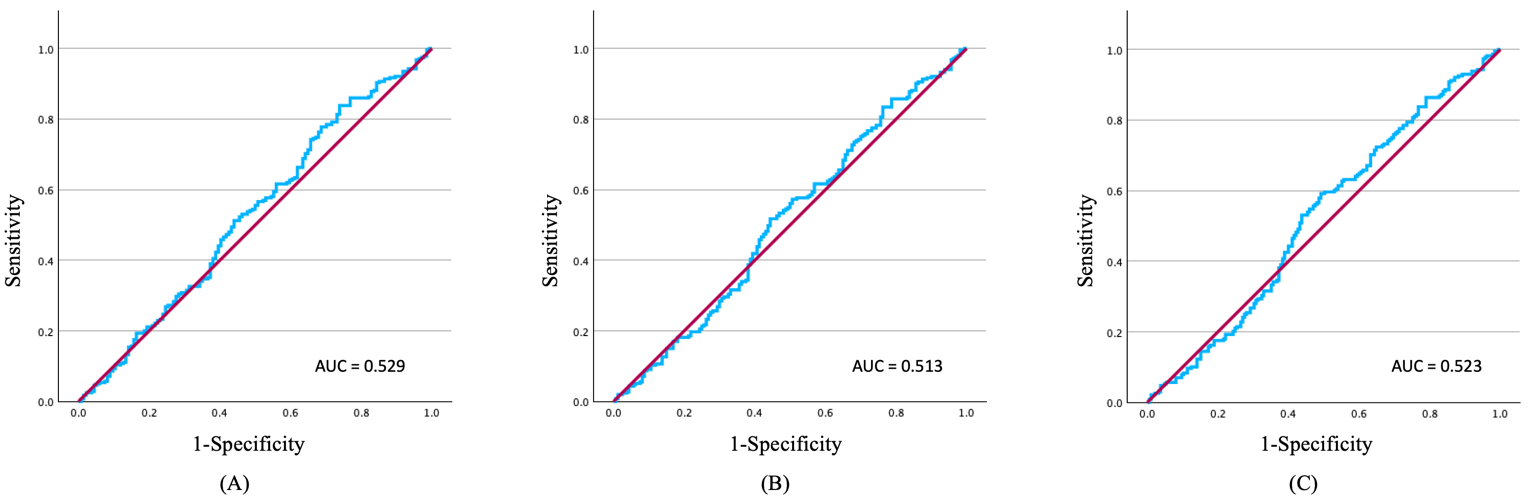


AUC, area under the curve.

**Supplemental Figure 4.** Subgroup analysis for (**A**) ICU, and (**B**) In-hospital all-cause mortality.


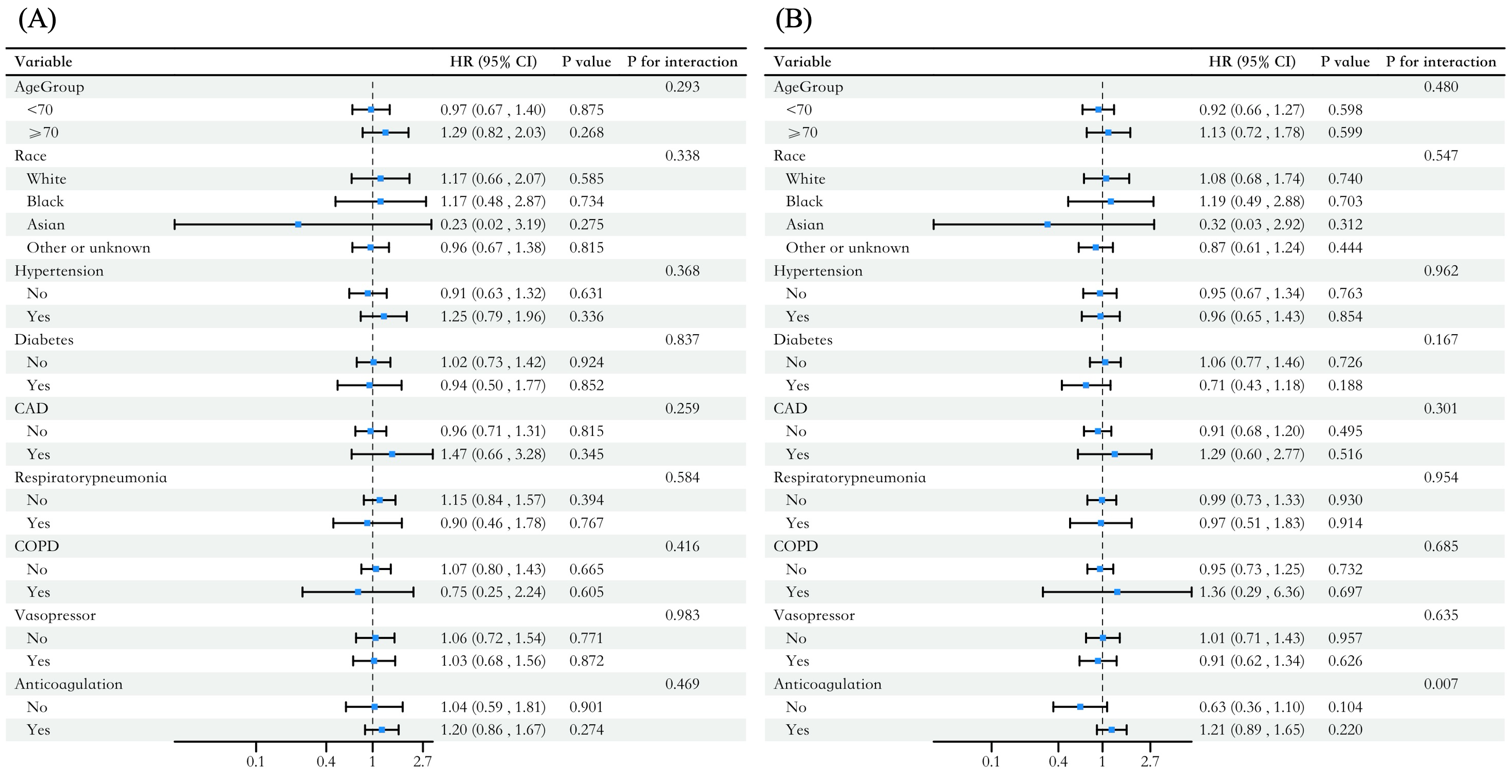


HR, hazard ratio; CI, confidence interval.
